# The Silent Aneurysm Behind the Hoarse Voice: Ortner's Syndrome

**DOI:** 10.7759/cureus.111979

**Published:** 2026-07-03

**Authors:** Jaisy J Vikkath, Rakshita Segar, Dhanya Velrajan, Surya TS

**Affiliations:** 1 Department of Otorhinolaryngology, PSG Institute of Medical Sciences & Research, Coimbatore, IND

**Keywords:** aortic arch aneurysm, cardiovocal syndrome, left recurrent laryngeal nerve palsy, ortner's syndrome, unilateral vocal cord palsy

## Abstract

Unilateral recurrent laryngeal nerve palsy presenting with hoarseness of voice is a common clinical condition with multiple etiological factors, most commonly malignancy, iatrogenic injury, and neurological disorders. Cardiovascular causes are relatively uncommon and are described under Ortner's syndrome, also known as cardiovocal syndrome. Here, we describe the case of a 76-year-old gentleman who presented with progressively worsening hoarseness of voice and aspiration of fluids for two months. Video laryngoscopy revealed left vocal cord palsy and a phonatory gap. Contrast-enhanced computed tomography of the neck and chest demonstrated a saccular aneurysm of the distal aortic arch with mural thrombus compressing the course of the left recurrent laryngeal nerve. A diagnosis of Ortner's syndrome secondary to a saccular aortic arch aneurysm was made. This case highlights the need to consider Ortner's syndrome as a possible diagnosis in patients presenting with new-onset hoarseness of voice, particularly those with underlying cardiovascular disease.

## Introduction

In 1897, Norbert Ortner published a report of three patients presenting with hoarseness of voice secondary to left atrial enlargement due to mitral stenosis, due to compression of the left recurrent laryngeal nerve [1]. This eponymous syndrome initially described hoarseness of voice exclusively caused by left atrial enlargement but later became synonymous with the cardiovocal syndrome proposed by Stocker and Enterline in 1958 to include recurrent laryngeal nerve palsy due to any cardiovascular pathology [2,3]. Compression of the recurrent laryngeal nerve (a branch of the vagus nerve) can cause vocal cord palsy, which leads to symptoms like hoarseness of voice, aspiration, and stridor. The left recurrent laryngeal nerve loops under the aortic arch before it ascends in the tracheo-esophageal groove, thereby making it susceptible to compression from any cardiac pathology. 

Initially, Ortner's syndrome only consisted of vocal cord palsy due to mitral stenosis; later, it went on to include conditions such as cardiomegaly, pulmonary artery dilatation, left atrial enlargement, and aortic aneurysm [4].

The case described below is that of a gentleman who presented with hoarseness of voice and difficulty swallowing but was found to have a thoracic aortic arch aneurysm. It highlights the importance of considering cardiovascular causes in the evaluation of unilateral recurrent laryngeal nerve palsy.

## Case presentation

A 76-year-old gentleman presented to the otorhinolaryngology outpatient department with complaints of hoarseness of voice and aspiration of fluids over the past two months. There was no history of throat pain, difficulty breathing, or painful swallowing. There were no signs of hemoptysis or loss of weight and appetite. He had undergone a percutaneous transluminal coronary angioplasty (PTCA) seven years ago. He was a smoker for 30 years but stopped 20 years ago (30 pack-years).

On examination, the patient was well-built, and his vitals were stable, with a pulse rate of 66 beats per minute measured on the right arm. His blood pressure was 130/80 mmHg in the right arm and 140/80 mmHg in the left arm. Videolaryngoscopy revealed left cord palsy with a phonatory gap and pooling of saliva in the left pyriform fossa (Figure 1). There were no mass lesions. Examination of the ears and nose was normal.

**Figure 1:**
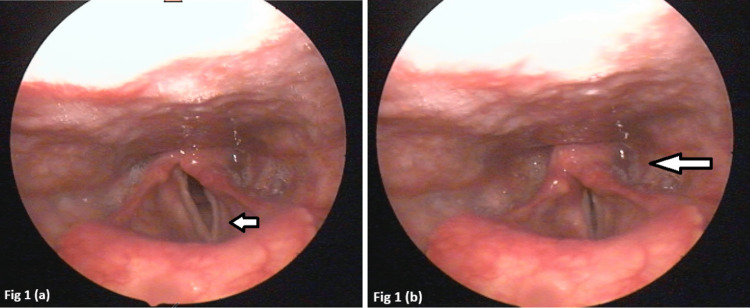
Videolaryngoscopy showing left vocal cord palsy (arrow, Figure 1a) with minimal pooling of secretions in left pyriform fossa (arrow, Figure 1b). Figure 1a is taken during respiration, while Figure 1b is taken during phonation.

Examination of the neck did not reveal any thyroid or lymph node enlargement. On auscultation, S1 and S2 were heard, with no abnormal sounds.

A complete hemogram, blood sugars, and renal profile were done, which were within normal limits. A contrast-enhanced computed tomography (CT) neck with chest was done, which was suggestive of a saccular aneurysm with a mural thrombus in the distal arch of the aorta, just after the thoracic origin of the left subclavian artery, with a lumen measuring 40 x 43 mm and the thrombus measuring 20 mm (Figure 2). The patient was diagnosed with Ortner's syndrome.

**Figure 2:**
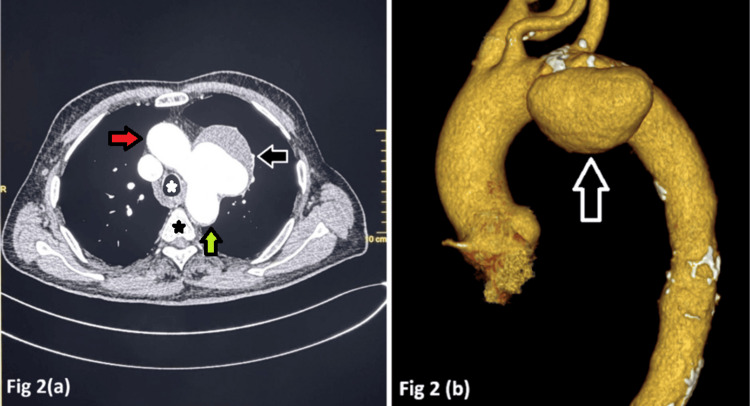
Contrast-enhanced computed tomography (CT) (a) and CT aortogram (b) showing a saccular aneurysm with mural thrombus in the proximal arch of aorta. White star, bronchus; black star, vertebra; green arrow, descending aorta; black arrow, saccular aortic arch aneurysm; red arrow, ascending aorta. CT: computed tomography.

Cardiology and cardiothoracic surgery opinions were obtained, and the patient was advised for hybrid aortic arch repair with debranching surgery. As the patient was not willing for surgery due to age constraints, he was started on metoprolol 12.5 mg once daily with a blood pressure target of 130/90 mmHg and was advised to repeat the CT aortogram after six months. However, he was lost to follow-up.

## Discussion

Unilateral RLN palsy typically presents with hoarseness of voice, with a minority presenting with cough with aspiration, difficulty swallowing, and weakness of voice. Most cases of unilateral RLN palsy worldwide are attributed to iatrogenic causes [5] like thyroid surgeries [6]. However, India deviates from this norm as malignancies are the most common cause of unilateral RLN palsy [7]. Cardiovascular diseases, like atrial enlargement due to valvular disease [8,9] and atrial myxomas (only one case has been reported in the literature so far) [10], were responsible for only a minority of cases.

Thoracic aortic aneurysms are focal or diffuse dilatations of the aorta that involve all three layers and are prone to tearing or dissection. Etiologies include old age, hypertension, atherosclerosis, smoking, and Marfan syndrome. As this patient has undergone PTCA previously, etiologies such as infection (mycotic aneurysms due to *Staphylococcus* infection), pseudoaneurysms (due to arterial injuries occurring during the procedure), and aneurysms arising in response to the stent have to be considered. Patients typically present with chest pain radiating to the back, breathlessness, myalgia, and syncope. In a few cases, due to mass effect, patients may even present with hoarseness of voice. This is the most common cause of cardiovocal syndrome, seen in 41% of patients [4].

The pathophysiology of this condition is due to the course of the left recurrent laryngeal nerve and its intimate relation with the aortic arch (Figure 3). The left recurrent laryngeal nerve branches from the left vagus nerve and enters the aortopulmonary window (between the pulmonary artery and the aortic arch), behind the ligamentum arteriosum. It then ascends through the neck and enters the trachea through the tracheoesophageal groove. However, 0.57% of people possess a “non-recurrent” laryngeal nerve, which innervates the larynx directly without entering the thorax [11].

**Figure 3:**
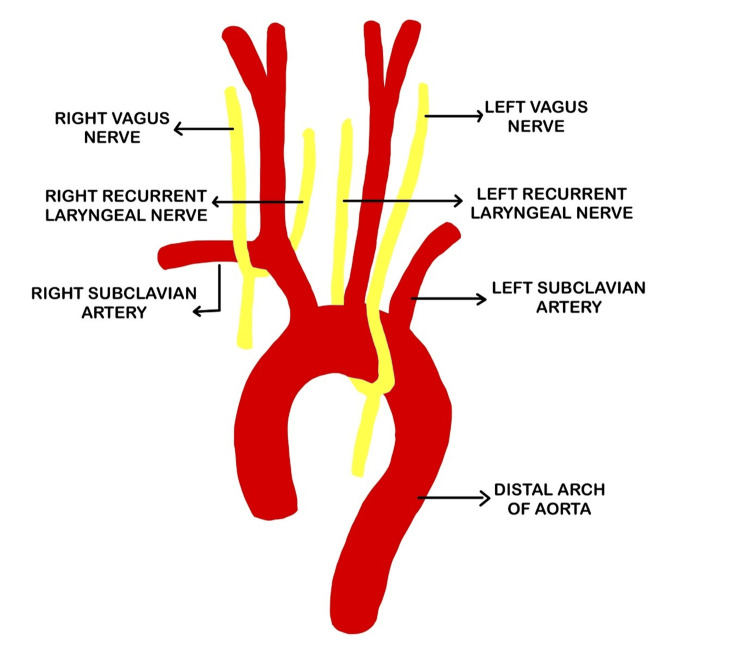
A schematic diagram of the proximity of left recurrent laryngeal nerve and the arch of aorta.

Partial or gradual compression of the nerve may not always result in hoarseness of voice; hence, many cases may remain undiagnosed.

## Conclusions

Ortner's syndrome is a rare cause of unilateral recurrent laryngeal nerve palsy. Thoracic aortic arch aneurysms may remain clinically silent and present solely with hoarseness of voice due to compression of the left recurrent laryngeal nerve. Early recognition and appropriate imaging are essential for establishing the diagnosis and preventing potentially life-threatening complications associated with aortic aneurysms. This case emphasizes the importance of a thorough evaluation of unexplained vocal cord palsy and highlights the need for multidisciplinary management in patients diagnosed with Ortner's syndrome.
